# Honoring the enslaved African American foremothers of modern women's health: Meditations on 40 years of Black feminist praxis

**DOI:** 10.1111/maq.12836

**Published:** 2023-11-27

**Authors:** Rachel Dudley

**Affiliations:** ^1^ Women's and Gender Studies, Affiliated Faculty in Africana Studies University of Toledo Toledo Ohio USA

**Keywords:** Black Feminism, Anarcha, Lucy, Betsey (and the unnamed others), Foremothers of American Gynecology, Gender & U.S. Slavery, Historical Anthropology, Cultural Memory & Healing Justice

## Abstract

This article analyzes 40 years of Black feminist scholarship, art, and grassroots activism dedicated to the lives and legacies of the “foremothers of American gynecology.” Infamously, in Montgomery, Alabama, between 1845 and 1849, up to 16 enslaved women were exploited at a backyard hospital, some subjected to surgical experimentation by Dr James Marion Sims. He was a famous and world‐renowned surgeon who died in 1883, with a reputation as “the father of modern gynecology.” Sims achieved the medical knowledge that catapulted him into American and European fame, using skills gained from the exploitation of the enslaved women in his early career. Famously, three of these women are referenced by their first names: Anarcha, Lucy, and Betsey. This research asks: how have these important figures been remembered in 20th and 21st‐century Black feminist scholarship, art, and grassroots community activism? Further, what are the broader impacts of this pathbreaking truth, reckoning, and reconciliation work?

## INTRODUCTION

This article documents nearly 40 years of Black feminist scholarship, art, and grassroots activism dedicated to the nonconsensual “foremothers of American gynecology.” These foremothers (of “modern women's health” more broadly speaking) include up to sixteen 19th‐century African American women who were enslaved in the American South. What happened to them represents a well‐known and symbolically important story in the history of medicine and slavery, medical ethics, exploitative research with human subjects, and reproductive injustices. These enslaved teenagers, from areas in and around Montgomery, Alabama, were subjected to years of medical abuse at Dr James Marion Sims’ backyard slave hospital. Some of the young women underwent numerous rotations of experimental vaginal surgeries, between 1845 and 1849 (Sims, 1884, 222–46). Famously, only three of the women were mentioned in Sims’ life‐writing, by their first names, as Anarcha, Lucy, and Betsey. Their exploitation allowed Sims to achieve international fame as a skilled surgeon and a revered pioneer in the field of modern American gynecology.

The young women endured a distinctively brutal and traumatic form of experimentation at the hands of Sims, in the age of 19th‐century scientific racism. He performed vaginal surgeries on the women, without anesthesia, to repair vesicovaginal fistulas (vaginal and rectal tears derived from the reproductive trauma of slavery, leading to incontinence, odor, discomfort, infection, and social stigma). Notoriously, Anarcha, from the large and well‐known Westcott plantation, experienced at least 30 surgeries.

**FIGURE 1:**
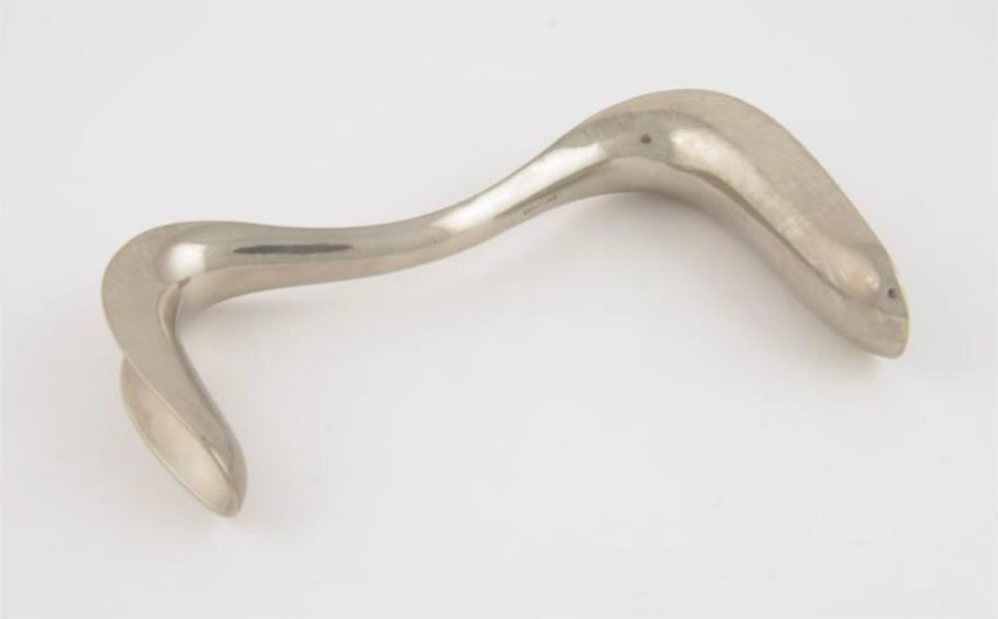
Science Museum Group. Sims double duck‐bill vaginal speculum, circa 1871. https://tinyurl.com/ynudbedw. Creative Commons License. Accessed February 13, 2023. [This figure appears in color in the online issue]

Referencing Katherine McKittrick, the backyard medical clinic served as a unique “cartography of struggle” for enslaved populations who feared Southern doctors and frequently underwent cruel procedures designed to advance scientific medical knowledge (Cooper Owens, 2017; Kenny, 2015; McKittrick, 2006, 8). Sims’ exploitation of the institution of slavery allowed him to develop a surgical cure for vesicovaginal fistulas, to publish the results in international medical journals, to create the Sims position, and to invent the Sims duckbill speculum, an instrument used in gynecology exams, as a medical technology (Figure 1).

This article presents a thematic review of Black feminist praxis about the 16 enslaved women's lives and legacies since 1985. Adding an important contribution to medical anthropology, this research provides *both* a Black feminist intellectual history *and* an arts‐based social movement history in this area. It traces the impact of successful Black feminist scholarly and grassroots interventions on the American medical, cultural, and political landscapes over a span of nearly 40 years. In so doing, this article shows how these efforts have culminated into a rich legacy of truth, reckoning, and reconciliation work.

In relation to my own positionality, I am a Black Women's Studies and Africana studies scholar of African American ancestry, specializing in medical history, feminist pedagogy, and interdisciplinary health humanities research. One of the great‐nieces of 19th‐century Southern suffragist and civil rights activist, Sara Dudley Pettey, I also have the benefit of a rich family legacy in grassroots advocacy, gender equity, and social justice work (see Gilmore, 1996). One of my great‐uncles, Edward R. Dudley, was a lawyer with the NAACP who worked alongside Thurgood Marshall as a civil rights activist. He also integrated US diplomatic embassies as the first African American ambassador in US history, appointed by President Harry S. Truman to serve as ambassador to Liberia. At the same time, together in 1949, my great grandparents—Alice B. Burton and Dr Dewitt T. Burton, MD—founded Burton Mercy Hospital in Detroit, Michigan. The hospital provided care for Black patients during the Black hospital movement in the era of racially segregated healthcare (see Northington Gamble, 1995).

Now a faculty member at The University of Toledo (a teaching‐centered, public research institution located in urban northwest Ohio), I research and teach about gender, race, and health equity issues. I began documenting Black feminist scholarly, grassroots and arts‐based interventions dedicated to the “foremothers of American gynecology,” beginning with graduate‐ level research at The Ohio State University, and later at Emory University (see Dudley, 2012, 2016, and 2021). I am also a proud member of the Black Feminist Health Science Studies Collective, whose mission is to “highlight the necessity of incorporating social justice into medical science” (www.blackfeministhealthstudies.com).

With my own positionality made clearer, the remainder of the article is grounded in Saidiya Hartman's *Lose Your Mother: A Journey Along the Atlantic Slave Trade Route* (2007) and in Deirdre Cooper Owens’ *Medical Bondage: Gender, Race and Slavery in the Origins of American Gynecology* (2017). I emphasize Cooper Owens’ framing of the enslaved women in this history as “medical superbodies,” juxtaposing this conceptualization with Hartman's concept of “the afterlife of slavery.” Significantly, Cooper Owens references the contradictions in the women's status as exploited and powerless “racial others” in 19th‐century, scientific racist‐patriarchal schemas, and their simultaneous status as highly trained healthcare workers in the first women's hospital in the country (2017, 108).

My research adds new dimensions to these pivotal works of Black feminist scholarship, contextualizing them within a larger 40‐year history of Black feminist praxis about the enslaved foremothers of modern US gynecology. The remainder of the article demonstrates how the enslaved women's legacies cut across various centuries, geographies, and knowledge‐communities—resonating with the cultural zeitgeist on a deeply symbolic level, well into the 21st century.

## METHODS AND METHODOLOGY

I used four methods for this research project. First, I conducted a thematic literature review of Black feminist intellectual thought, specifically about Anarcha, Lucy, Betsey, and the unnamed others, published from 1985 to the present. This period was chosen as a starting point because it represents an important moment in second wave, US Black feminism, alongside the development of Africana Studies paradigms. Such an approach represents a distinct genealogy of thought, involving multiple disciplines and generations of scholarship, including the medical sciences, social sciences, and the health humanities.

Second, I carried out archival research at the Louis Round Wilson Library at the University of North Carolina at Chapel Hill. In this article, I analyze two newspaper clippings from this archive, showing how they are representative of memorialization efforts dedicated to Sims following his death in 1883. The clippings serve as a microcosm, allowing us to pinpoint the moment of historical erasure for the enslaved women and the ways in which hagiographic depictions of Sims started taking shape after his death. This material provides important theoretical framing because it highlights what Black feminist praxis has been responding to, writing about, and organizing against for so long.

Third, I include excerpts from an interview with Ebony Golden, a community‐based performance artist and organizer in Harlem, New York, who founded the Betty's Daughter Arts Collaborative (BDAC). The collaborative staged an important “ring‐shout for reproductive justice” in front of a statue of Sims in 2011. In telling this story as a Black feminist intellectual and arts‐based social movement history, the inclusion of Ebony Golden is important in representing a grassroots profile. BDAC's street‐performance work preceded the 2018 removal of a statue of Sims from New York's Central Park. A BDAC performance ensemble known as *Body Ecology* created a symbolic watershed moment in street‐level, arts‐based activism memorializing the foremothers of American women's health. They did so to recast a narrative in front of the statue, to raise public consciousness, and to enact positive social change at a local level, linking the history to contemporary reproductive justice struggles for Black, Indigenous, and other people of color communities (BIPOC).

Fourth, I provide and contextualize relevant excerpts from “Victory Beyond Sims: A Virtual Community Report Back,” an online event hosted and recorded by the New York Academy of Medicine, where community members spoke to acknowledge a decades‐long effort to remove and replace the Sims statue from Harlem, New York. The event featured key voices in the effort to raise awareness, including members of the local Committee to Empower Voices for Healing and Equity, writer/medical ethicist Harriet Washington, and public sculptor Vinnie Bagwell. In the last section of the essay, I bring readers up to the present historical moment by discussing the community‐based, grassroots work taking place in Montgomery, Alabama—spearheaded by artist and community organizer Michelle Browder. In addition to organizing a series of annual conferences around this history, Browder has produced new portraiture dedicated to the enslaved women. Further, she has created *The Mothers of Gynecology* monument and garden, which was unveiled in 2021, with plans to construct a $5.5 million dollar museum and medical center for underserved communities. Taken together, this methodological approach shows how Black feminist praxis has sustained an emphasis on cultural memory, ancestral legacy, as well as reproductive and healing justice, over time.

## FRAGMENTED LIFE SKETCHES OF ANARCHA, LUCY, BETSEY, AND THE UNNAMED OTHERS

**FIGURE 2:**
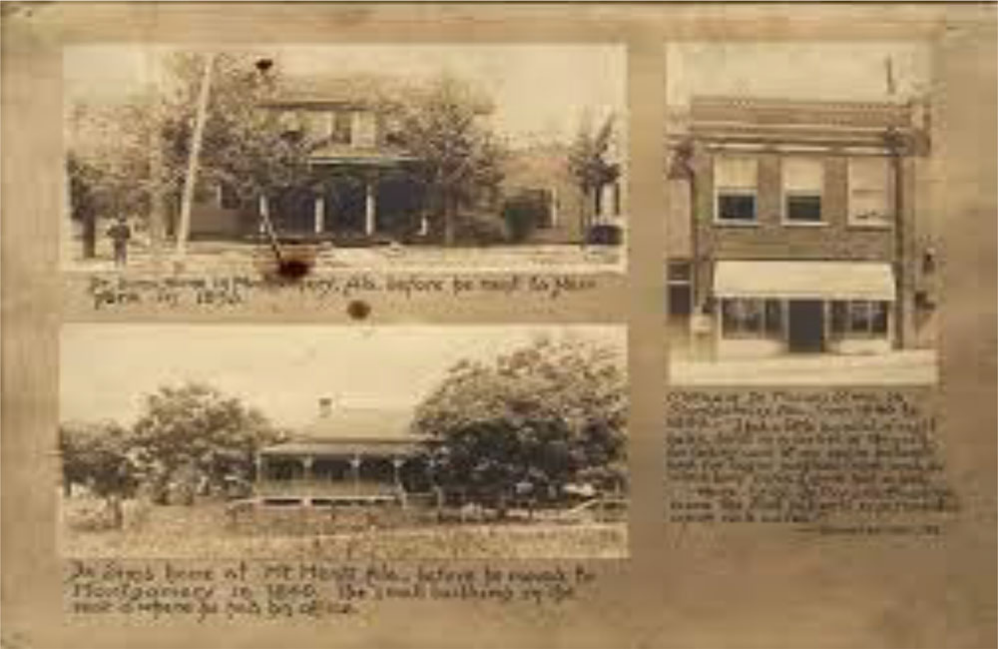
Image of homes of Dr James Marion Sims in Montgomery (upper left) and Mount Meigs, Alabama (lower left), and his clinic in Montgomery (right). The photograph is copyright of the Alabama Department of Archives and History, Photographs and Pictures Collection. [This figure appears in color in the online issue]

In the mid‐19th century, Alabamian slaveholders were eager to find a medical fix for the fistula condition because it threatened the profit economy of slavery (see Schwartz, 2010). Fistulas were an indicator of the violence Black women experienced under slavery—resulting directly from bodily trauma (McGregor, 1998, 68). This form of bodily trauma can be related to sexual violence, use of forceps and/or other medical instruments and/or numerous, particularly difficult labors. The women in this story were in their late teens and may have been mothers of other children before transport to Sims’ hospital in 1845 (Figure 2).

Betsey was 18 and recently married, though her husband's name is unknown. Lucy was the youngest of the three at 17. Anarcha was 19 and mourning a recent stillbirth. In the summer of 1845, Sims wrote to surrounding plantation owners to inquire about any cases of fistula that might have been, “tucked away in the surrounding countryside,” requesting to keep the women for experimentation if their slave holders agreed to pay for taxes and clothing. He explains:

I ransacked the country for cases…[a]nd it ended in my finding six or seven cases of vesico‐vaginal fistula that had been hidden away for years in the country because they had been pronounced incurable (sic). I went to work to put another story on my hospital, and this gave me sixteen beds; four beds for servants, and twelve for the patients. (Sims, 1884, 236).

Upon train and/or horse carriage transport to Sims’ 16‐bed slave hospital, located behind his clinical practice, these young women were separated from their potential surviving children, their loved ones, and their broader communities, though they had each other. In the first couple of years, Sims had the assistance of other Montgomery‐area medical students and doctors.

Eventually, the other medical men stopped coming, believing Sims would not be successful in his experimental exploits. Sims then started to train the women in their own nursing care. They were administered opium for pain, for bowel blockage and for compliance (hence the very real possibility of narcotic substance addiction, which has been emphasized in Black feminist conceptualizations of this history). However, they were not administered ether as anesthesia, which was slowly coming into broader use after its discovery by a Boston dentist named William Morton in 1846 (see Pernick, 1983).

Given that there were 16 beds and only three names in Sims’ writing, this research holds space for not only Anarcha, Lucy, and Betsey, but also the unnamed others. Including them in this research represents a nod to other marginalized stories that have been lost in the voids, gaps, silences, and absences in the historical record. In addition to Anarcha, Lucy, Betsey and the other unnamed women, Sims also experimented on enslaved men and children at other times in his early career, including in dentistry and for lockjaw (Sims, 1884, 19). Thus, the phrase “the unnamed others” is a powerful symbolic and representative placeholder.

Recent historical scholarship demonstrates that after the experimental series, Anarcha (also spelled Annacay) married a man named Laurenzi Jackson, and she died in 1869 at the age of 46, buried next to her husband (Hallman, 2023, xvi). Anarcha Jackson knew romantic love, and she lived through the abolition of slavery. Importantly, the story of what happened to her, and the others, loomed large in the medical legacy of Sims and needed to be addressed by the time of his death in 1883. Yet the enslaved women are ghost‐like (both there and not there) within 19th‐century newspaper memorialization. The question Sims’ contemporaries had to grapple with was what to say (or not say) about the enslaved people and the role of slavery in that infamous, backyard Montgomery hospital—following the Civil War and the Reconstruction Era.

The following excerpts, found in the J. Marion Sims Papers at the Louis Round Library, Box #666, are from two unknown newspapers. They were published among hundreds of national and international memorialization dedications to Sims following his death:

In 1845, also, he became especially interested in the subject of vesico‐vaginal fistula, a disease which had hitherto been regarded as incurable. Beginning a course of experiments in regard to it, he supported a private hospital in Montgomery and after four years of unwearied application, met with complete success.

His great discovery, the one by which his name will always be memorable in the annals of surgery, was carefully thought out beforehand; it was prosecuted with long delays and disappointments, and at last as unswervingly carried out, and with perfect and individual success, as Columbus's discovery of the New World. This discovery, that of the cure of a grievous and previously incurable complaint (vesico‐vaginal fistula), and the subsequent introduction of silver sutures in surgery, are the main triumphs of the eminent career that is now closed.

In these clippings, the enslaved women have vanished from direct mention. Further, the comparison of James Marion Sims to Christopher Columbus blatantly situates access to Black women's bodies within geographies of 19th‐century white masculine exploration, and within paradigms of colonialist medical knowledge production. This microcosm of newspaper memorial dedication allows us to examine the cultural, historical, and medical hagiographies (saintly representations) of Sims and the politics of erasure that Black feminist scholars have been deconstructing for decades.

## YEARS OF BLACK FEMINIST SCHOLARSHIP: NAMING AND HONORING THE NONCONSENSUAL FOREMOTHERS OF MODERN WOMEN'S HEALTH

The literature review below analyzes 40 years of multi‐disciplinary Black feminist scholarship published about the foremothers of American gynecology and modern women's health. I demonstrate how this scholar‐activism signifies an unbroken thread of truth and reconciliation work. The review is organized into the following themes: cultural memory, ancestral legacy, and healing justice.

### Cultural Memory

Beginning with the publication of Diane Axelsen's “Women as Victims of Medical Experimentation” in 1985 and Durrenda Ojanuga's “The Medical Ethics of the “Father of Gynaecology,” Dr J Marion Sims” in 1993, Black feminist scholars have written to preserve the cultural memory of the enslaved women and to place their stories in larger bio‐ethical, political, and socio‐historical contexts (Axelsen, 1985; Ojanuga, 1993). In doing so, Black feminist scholars have also made broader points about power relations and the racialized and gendered dynamics of public memorialization.

In this case, “cultural memory” refers to a collective effort, dedicated to the women in this history, to document *their* legacies through cultural symbols and material culture artifacts in the written and public domains. We can see this, for example, in the work of five highly decorated African American US poets. These poets include Nikky Finney, with her poem “The Greatest Sideshow on Earth” in *The World is Round* (2013); scholar‐poet Bettina Judd, with her various publications in this area, including her book *Patient. Poems*. (2014); Kwoya Fagin Maples, with her book, *Mend Poems* (2018), and Dominique Christina, with her book *Anarcha Speaks: A History in Poems* (2018). These works emphasize the cultural memory aspects of the enslaved women's legacies through art. Notably, Judd begins her poem “After Memory” with a nod to the prolific African American poet Lucille Clifton (1934–2010). The epigraph of Judd's poem cites Clifton's “Why Some People Be Mad at Me Sometimes,” by excerpting the famous and apropos line: “i keep on remembering mine” (Clifton, 1987, 6).

Over the years, Black feminist artists and scholars have remembered (and re‐ remembered) the enslaved women's legacies through creative endeavors, by writing extensively, and by speaking back to reductive criticisms and specious arguments from Sims’ apologists.

These arguments portray Sims as simply a man of his times and claim that focusing on the enslaved women, forced opium addiction, the impossibility of consent, exploitation, and torturous abuse obscures Sims’ useful contributions to medical knowledge (Vernon, 2019; Wall, 2006; and 2018; Natur*e* editorial with unidentified author, 2017).

Black feminist scholars have written against this vein of thinking, not to dismiss the gains of modern medical knowledge, but to properly contextualize them in relation to social power arrangements, structural violence, and 19th‐century patriarchal scientific racism. This vein of Black feminist scholarship also includes works about the ethics of statues dedicated to Sims and the politics of monument culture in a 21st century context (Green, 2017, Washington, 2006, and 2017). The groundbreaking work of historian and medical ethicist Harriet Washington highlights histories of medical abuse against African Americans, from colonial times to the present. Her 2006 book, *Medical Apartheid*, begins with the ethics of public monument dedications to Sims, and the cultural erasure of the enslaved women.

There has also been important cultural work by allies in feminist performance studies, including Terri Kapsalis’ “Mastering the Female Pelvis: Race and the Tools of Reproduction,” published in Black feminist scholar Kimberly Wallace‐Sanders’ edited anthology, *Skin Deep Spirit Strong: The Black Female Body in American Culture* (2002, 263–300). The pathbreaking anthology in Black feminist studies also includes “The Body Politic: Black Female Sexuality and the Nineteenth‐Century Euro‐American Imagination,” by Beverly Guy‐Sheftall, Anna Julia Cooper Professor of Women's Studies and Director of the Women's Research and Resource Center at Spelman College (2002, 13–36). These chapters represent significant works in framings of Black female sexuality, medical history, and international cultural representations in the 19th century (also see Gilman, 1985).

Additionally, there has been Black feminist work in the form of public scholarship, including a 2015 National Public Radio interview on the show *Hidden Brain* called, “Remembering Anarcha, Lucy, & Betsey: The Mothers of Modern Gynecology” (2017). Hosted by Shankar Vedantam, the show featured Bettina Judd, PhD, and Vanessa Northington Gamble, MD, an internationally esteemed African American physician, who chaired the 1996 Tuskegee Syphilis Study Legacy Committee. Here, Judd and Northington Gamble claim cultural space for the enslaved women as important Black ancestors, by publicly naming them as the “mothers of modern gynecology,” acknowledging them as extremely influential (and nonconsensual) figures in modern women's health.

I have also published an article in a *Humanities* special issue on social memory about placing this history in the context of health humanities research (Dudley, 2021). The article explores the concept of *poetic ancestral witnessing* in this history, through readings of the African American women's poetry mentioned above. The *Humanities* article includes results from an interview with Dr Judd, a Black Feminist Health Science Studies Collective member, analyzing her work in relation to Black feminism, art, and cultural memory. In addition to cultural memory, the idea of honoring ancestral legacy also emerges as an important theme from 40 years of Black feminist scholarship on Anarcha, Lucy, Betsey, and the unnamed others.

### Ancestral Legacy

Ancestral legacy, spirituality and the sacred represent important themes in Black feminist scholarship in general (see Alexander, 2005; Evans, 2021; Holland, 2000; Pauline Gumbs, 2016). In relation to this specific history, Black feminist scholars have been especially interested in reclaiming space for the enslaved women's possible interior lives, as important African American ancestors. This scholarship also emphasizes the impossibility of consent and the diminishment of the enslaved women's personhood and pain, highlighting how the false belief that Black people had a higher pain tolerance was used to justify conditions of slavery (see Davis, 1983).

In Black feminist scholarship, the enslaved women's legacies are often discussed in the context of contemporary US healthcare disparities regarding race, pain, cultural competency, and gendered/racial empathy gaps. In this sense, sharing the enslaved women's stories creates affective space for other stories of Black women's experiences of contemporary medical encounters and injustices to be documented. Reading some of these stories can be productive for health humanities, legal, and medical professions training (see Alford Washington, 2009). This vein of Black feminist scholarship negotiates ancestral legacy and plays with allegories of hauntings and specters, asking, what continues to resonate about this history for Black communities in relation to 21st century healthcare, and why?

This includes publications derived from experiences in the *Anarcha Project*, spearheaded by disability studies scholar and ally Petra Kuppers (Kuppers, 2008; Steichmann, 2008). The project included numerous community‐centered readings and performances throughout the country from 2006 through 2007. The point was to examine disability histories alongside African American histories through the stories of Anarcha, Lucy, Betsey, and the unnamed others. At least two notable Black feminist scholarly works were derived from participation in the *Anarcha Project*, including “With Anarcha: A Meditative Diary on Personal Healing and Touching History Through Performance Practice,” by Aimee Meredith Cox, and “In the Shadows of Anarcha: Race, Pain, & Medical Storytelling,” in *This Suffering Will Not Be Televised: Black Women and Sentimental Political Storytelling*, by Rebecca Wanzo (Meredith Cox, 2008; Wanzo, 2009). These important works (plus dissertation interviews with both scholars and grad school mentorship by Dr Wanzo), played significant roles in my early interest in this research area.

Adopting a first‐person perspective, these scholars discuss participation in the *Anarcha Project*, reflecting on personal and social healing, and traumatic encounters within the US medical industrial complex. These works are important in highlighting the processing of historical and contemporary harms related to this history, through cultural performance, scholarship, and medical storytelling. In discussing the enslaved women's ancestral legacies within medicine, these authors acknowledge their personhood and their exploitation, tracing historical and contemporary issues with the dismissal of Black pain, stereotyping, and conscious and/or unconscious biases. Moreover, these publications help readers with meaning‐making in relation to this important history—while gesturing towards personal and cultural healing.

### Healing Justice

The concept of *healing justice* comes from the work of Black feminist scholar and grassroots leader Cara Page, and the Kindred Southern Healing Justice Collective (Page and Raffo, 2016). They have articulated healing justice as: “a political strategy…[t]o intervene and respond on generational trauma and systemic oppression and build community/survivor led responses rooted in southern traditions of resiliency to sustain our emotional/physical/spiritual/psychic and environmental wellbeing” (http://kindredsouthernhjcollective.org). Through this framework, we can understand how Black feminist scholars have imaginatively engaged in prolonged truth, reconciliation, and healing justice work in relation to the history of slavery and the foremothers of American gynecology/modern women's health.

Of course, Columbia Professor of English and Comparative Literature Saidiya Hartman is one of the most preeminent Black feminist scholars to publish on gender, race, and US slavery in the 21st century. In her works, including *Scenes of Subjection: Terror, Slavery, & Self‐Making in Nineteenth Century America* and *Lose Your Mother: A Journey Along the Atlantic Slave Route*, she famously discusses how the “racial calculus” established under the logics of slavery continues to devalue Black life, well into the 21st century (see Hartman, 1997, 2006, 2007, and 2021). This devaluation exists affectively and materially, and it can be measured in many ways, including (for the purposes of this context) appalling inequalities in healthcare statistics, and in the structural violence of the medical industrial complex on Black bodies. The legacies of slavery also exist in relation to reproductive justice concerns for Black women, particularly disparities in Black maternal and infant health outcomes (Cooper Owens & Fett, 2019; Hammonds & Reverby, 2019).

In conceptualizing the term “medical superbodies,” history of medicine and medical humanities scholar Deirdre Cooper Owens helps us understand how Sims (and his 19th‐century colleagues) exploited enslaved women's reproductive laboring bodies—both on the medical exam table *and* in the medical clinic as trained nurses (Cooper Owens, 2017, 7). Black feminist framings of modern gynecology's development help us to see not only the continuing legacies of the enslaved women in this history but also the challenging ways in which the remnants of slavery and colonial medicine are still evident in our neo‐colonial healthcare systems (Cronin, 2020).

Cooper Owens focuses on the interplay of 19th‐century scientific racism, white male medical doctoring, and the management of Black women's reproduction within the institution of slavery. Further, she shows how large‐scale medical exploitation by acclaimed doctors, including Sims, and their subsequent publications in international medical journals, directly facilitated the development of gynecology as a Western, scientific, medical specialization by the 1870s.

In this vein of historical reclamation and healing justice work, the newly established Black Feminist Health Science Studies Collective (BFHSS, http://www.blackfeministhealth.com) published an open letter to the editors of *Journal of the National Medical Association* in 2019 (Bailey, et al 2019). The letter was a response to a review published by the journal defending monuments to Sims, titled, “J. Marion Sims MD: Why He and His Accomplishments Need to Continue to be Recognized, a Commentary and Historical Review.” In the open letter, the BFHSS collective included a list of works that could have been cited by the author, imploring the editors to publish the letter in full and to engage a more diverse group of reviewers before publishing similar works again. In this instance, the collective drew attention to the ways in which certain repetitive claims extend the women's original exploitation and willfully dismiss the history of Black feminist praxis in this area.

These efforts have helped shape the intellectual and cultural milieu, which influenced The American College of Obstetricians and Gynecologists to publish their article Joint *Statement: Collective Action Addressing Ra*ci*sm* (2020). The statement was written in the wake of the national reckoning with systemic racism, following the murders of George Floyd and Breonna Taylor at the hands of law enforcement in the United States. It includes 24 of the leading professional associations in gynecology and obstetrics, all acknowledging the contributions of Anarcha, Lucy, and Betsey and vowing to address current, systemic inequities in medicine. The joint statement affirms a commitment by the profession to eliminate disparities in women's healthcare through the following actions: collaboration, education, recognition, scholarship, research, publication, guidance, inclusive excellence, caring for patients and communities, as well as policy & advocacy work (The American College of Obstetricians & Gynecologists, 2020, 2).

## BLACK FEMINIST RESPONSES IN ART AND GRASSROOTS COMMUNITY ACTIVISM

I now turn to Black feminist arts‐based and grassroots, community‐centered activism, which allow us to highlight connections between art, representation, historical memory, and the present. For example, American performance artist and sculptor Doreen Garner has created an artistic performance piece called, “Purge,” which address gritty histories of sexism, racism, and medical trauma by playing with themes of gazing, butchering, inspecting, and classifying.

Another significant example of arts‐based direct action in this area, The Betty's Daughter Arts Collaborative (BDAC), was founded by Ebony Golden in Harlem (bettysdaughterarts.com/about‐bdac). Ebony is an artist, scholar, grassroots organizer, and force of nature. The performance ensemble put together “ringshouts for reproductive justice” in front of the Sims Statue in New York in 2011. The ringshout for reproductive justice street performance was in direct response to a series of anti‐abortion billboards that went up around the area, targeting Black women's reproductive, bodily autonomy.

BDAC wanted to shed light on long histories of laws, campaigns, institutions, and policies, attempting to control Black women's bodies and reproduction (see Ross & Solinger, 2017). They engaged in a methodical and organized effort, devising a Facebook campaign meant to express outrage and offer talking points for people doing social justice work who were interested in speaking out against the billboard campaign. In direct response to these attacks, which targeted African American women, organizations such as the Trust Black Women Campaign began mobilization efforts. In solidarity with these efforts, BDAC began planning ringshouts for reproductive justice in strategic public locations across the city, including the “Swing Low” Harriet Tubman memorial statue created by Alison Saar in Manhattan and the James Marion Sims statue in Harlem. A *ringshout* is a circle gathering rooted in Black diasporic ancestral traditions, which involves ritual, song, mourning, healing, togetherness, movement, poetry, rhythm, and music, as well as testimony and ancestral connection. In Ebony Golden's words: “[a] ringshout is a method for praise and worship. In the ringshout, people sing, dance, testify. Usually, the songs are led but there is time for each person to speak or sing.[..] The idea is that the circle is sacred and when those join in the circle, they harness an energy and power to manifest what they choose” (Golden & Dudley, 2013).

I interviewed Golden on Skype in 2013. The interview provides both a snapshot in time and a representative example of the kinds of successful grassroots work on the legacy of the foremothers of gynecology that has been ongoing for decades now. In the interview, I asked her about the role of womanist performance art and storytelling, the continuing relevance of this story for Black people in America, and the role of direct arts education and community‐building. With her verbal informed consent, I am grateful to include Ebony Golden's voice here, edited for clarity and context. First, I have excerpted her answers to questions about art‐based interventions and community‐building. Golden and other Body Ecology members were interested in emphasizing the enslaved women's ancestral legacies as well as notions of public artistry as an extension of sisterhood, healing, survival, and community:

**Ebony Golden**: Performance art is a way in which we are building. It's not just about theatrical performance, it's not just about a product. A big part of what we do happens for audiences and participants, but a big part of what we do involves some things that people never see. The real performance work is about sisterhood, thinking about what it takes for Black women to sustain community, and that is where in my mind liberation is seeded. And where it is born is in community, where black women can come together and heal and trust and talk, work, grow, plan, and eat and love, and do nothing but look at each other or look out the window…[W]e would not be able to perform work on the streets where we could possibly get arrested if we did not have a real practice of sisterhood. And it's not easy; it's messy, but for it to be linked to local social justice work, you must have a real connection, so you use the energy or fire that that creates and then make a theater piece.

Here, we can think of Black feminism and ancestral legacy in the sense of gathering in the memory of ancestors and reclaiming cultural spaces to remember them, to heal and to make more poignant points about current social justice struggles. This works affectively, geographically, and visually in stunning ways, in relation to street performance. Several times, Golden articulated the womanist approach of the group. So, I asked her to define the relationship of their work to Alice Walker's foundational articulation of woman of color feminism, in her famous essay, “Womanism.” The field‐defining essay appeared within Walker's larger book, *In Search of Our Mothers Gardens: Womanist Prose* (Walker, 1983). In the following interview excerpt, Golden discusses her take on womanist performance practice:

**Ebony Golden**: All our work is very much rooted in womanist performance methods. Womanist performance is performance that explicitly comes from a Black woman's sensibility and way of being in the world and way of engaging the world. A quick and dirty list of womanist methods we used in that performance…before any art is made, sisterhood is the foundation. The sisterhood is the core and when that's shaky, shifty, and shitty, so is the art. Number 2, being really hip to what's happening with black women outside of our selves. What's on the emotional, political, economic map for black women in Atlanta, in Oakland, in Philly. What's going on with the media, how are we being portrayed in mainstream media and virtual circles, so we have to do a check in.

Number 3, spiritual practice is critical and key, and this is not to take the place of religion. In terms of spiritual practice Body Ecology is all over the map and some of us don't identify as religious but we all understand spirituality as being a part of womanism…our ways of conjuring, weaving praying and meditation, lovemaking, cooking, humming, rocking, even looking at each other (laughter). All of that is spiritual practice and that is key especially when you get into ideas about how a character would be in the world…we go with how we would be in the world. Another aspect I would say is non‐hierarchical art making.

Once we get into the creative lab, we are all equally responsible for making it work. It's not about just doing what a director tells you to do. It's about a collaborative space.

Number 5, we have to accept and practice inclusivity…because all Black women are not the same, some of us are straight, queer, Christian, Muslim…we don't try to be all alike and celebrate difference in the art itself. Food is actually really, really important; it's an important aspect of communion. The practice of breaking bread is important…it's about “I love you; I want to make art with you, and I want you to be well.” It also requires a clear understanding of history. It's about black women being central to the story.

Womanist performance is a healing poetics that generates creativity, community, and a space for transformation and possibility.

The Afro‐futuristic vision in Golden's response is noteworthy. In this sense, the idea of ancestral legacy is about Black freedom struggles today having connection to “a clear understanding of history,” and a connection to community as a basis for ongoing social transformation.

In the interview, I also asked Ebony to explain why she chose to do a ring‐shout for reproductive justice in front of the Sims statue in Harlem, back in 2011.

**Ebony Golden**: We felt like we had to do the ringshout there in front of the Sims statue. We used it as a muse. We used the story of legacies of trauma of that, and we were thinking about performance as a type of mapping. So, we were thinking about how to map something different on top of a space that represents trauma and horror for Black women, our bodies, and experiences. So, it was our approach in doing this really was a way to bring ritual and inspiration and to bring contemporary analysis to a site that represented so much pain and trauma. Literally, people in a ringshout move counterclockwise, there's a leader who calls out the song and the community of ringshouters respond…[S]o, the ringshout is Afro‐futuristic, it's about using art to think forward about Black survival (sic).

It is fascinating to note Ebony's comments on playing with time and the Afro‐futuristic vision in her articulation of the ringshout. Her answer also expressed the collective's emphasis on bringing ritual and inspiration to the space and *remapping* something different on top of trauma and exploitation.

Finally, Ebony offered her perspective on how the story of Anarcha, Lucy, Betsey and the unnamed others continues to resonate in the 21st century context:

**Ebony Golden**: Right now, the Black woman's body is a microcosm of the police state. One of the primary battlegrounds in this country is over what it means to have freedom, what it means to have civil rights, privacy, control over your own body. So, this history, and his experimentation, is directly linked to what's happening now. Now it looks different because right now we have issues around food security, who gets to have babies, and who does not or access to reproductive healthcare. I think that Black women are very visible right now, but combined with the extreme ways in which we're invisibalized (sic) politically and in terms of media representation. So, these things haven't really changed, I think that might be saying a lot, but I don't think they've really changed. So just as important as it was then for people to be fighting and resisting and doing what they did to reclaim and recoup—coming up with herbal remedies, finding ways to do stitch work, et cetera—resistance work is just as important now.

Here, Golden mentions key issues of nutrition and food insecurity, reproductive justice, police and state violence, and the meanings of freedom and bodily autonomy and controlling media images of Black women (see Hill Collins, 1990). For Golden, to say that things have not really changed is not to say that Black women are still enslaved or facing the specific kinds of medical trauma endured under Sims. The point, instead, is that the afterlife of slavery still haunts the American imaginary and can be traced by concrete, material disparities in wealth, housing, nutrition access, education, employment, incarceration, policing, and in health outcomes.

### East Harlem, New York Preservation Inc. Virtual Community Report Back and Michelle Browder's More Up Campus in Montgomery, Alabama

The East Harlem Preservation Inc., led by community member and ally Marina Ortiz, sustained a 12‐year grassroots effort in Harlem, New York for the removal and replacement of Sims’ statue in Central Park. Through a multi‐racial and multi‐coalitional sub‐committee to empower voices for healing and equity, they coordinated with other community‐based organizations, cultural institutions, city agencies, and elected officials. During the recorded virtual event “Victory Beyond Sims: A Community Report Back,” James C. Horton, the vice president of education and engagement at the Museum of the City of New York, interviewed medical ethicist Harriet Washington (Victory Beyond Sims: A Community Report Back, 2021). To start the interview, Horton asked Washington, “what motivated you or inspired you to include J. Marion Sims statue in your book, *Medical Apartheid?*”

In the interview, Washington reveals her shock after learning from Dr. Sims’ own writing that everything she had learned about him was a lie. She explains: “*He was not a benefactor. He had not gotten these women's permission. These women were not patients, but they were subjects locked in a laboratory. So, when I learned that, it completely changed how I was going to treat him in the book.”* As noted above, Washington's 2006 book became a pivotal moment in *reckoning* with the enslaved women's lives and legacies on a national scale; it also helped usher in a new wave of Black feminist scholarship and grassroots action.

The community report‐back also acknowledges the renowned sculptor artist from Yonkers, New York, Vinnie Bagwell, with an interview (the event itself was named after Bagwell's proposed sculpture). In 2019, Bagwell's proposal, “Victory Beyond Sims” was selected by the local community in Harlem to replace the Central Park Sims statue. Bagwell is currently in talks with the Cultural Affairs Department regarding commissioning her work. The removal of the Sims statue in 2018, Bagwell's proposal, and the online event represent pivotal moments in acknowledging the enslaved women's lives and legacies, within public cultural memory. Since this story spans so many temporalities and localities, it is important to examine the local work happening at the geographic heart of this history in present‐day Montgomery, Alabama.

The More Up Campus non‐profit organization in Montgomery, spearheaded by Michelle Browder, has initiated several phases of memorialization efforts dedicated to the foremothers of American gynecology (http://www.anarchalucybetsey.org). So far, these efforts have included a *Remembering Anarcha* documentary starring *Browder*, two “Anarcha, Lucy, Betsey” conferences facilitated by The More Up Campus, plus a public monument and park dedicated to the enslaved women.

*The Mothers of Gynecology* monument was designed and created by Browder and placed within a couple of miles of the Alabama State House (which displays a concrete and bronze statue dedicated to Sims, erected in 1939). Unveiled in 2021, with funding from grants provided by organizations such as the Southern Poverty Law Center, *The Mothers of Gynecology* monument consists of three tall metal sculptures representing Anarcha, Lucy, and Betsey. These elevated figures force viewers to look up in reverence. Cut off at the knees and depicted as armless, they are each nude, and the statues incorporate glass shards and found metal objects (nails, spoons, bike chains, scissors, needles, and speculums), which are representative of the indignity and torture they endured. Notably, the statue in the middle depicts Anarcha with a hole in place of her womb.

**FIGURE 3:**
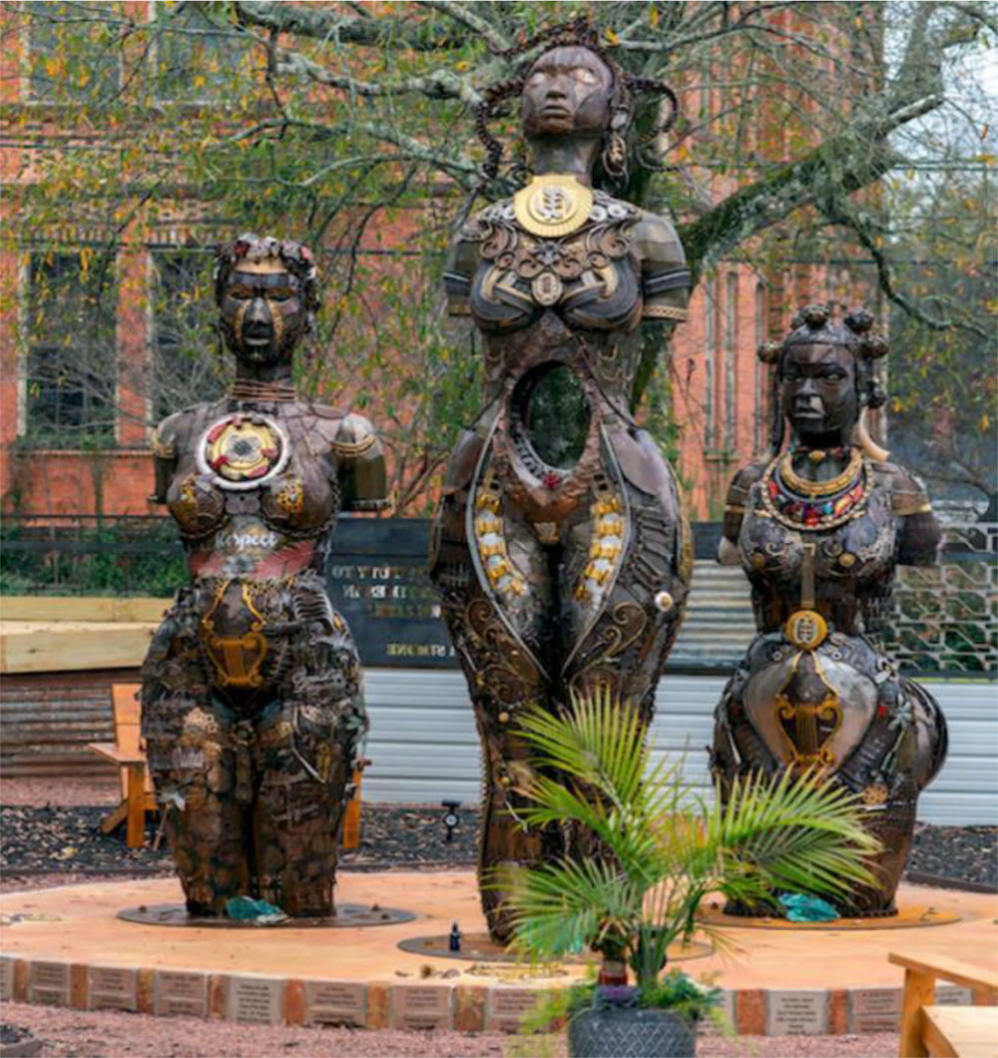
Michelle Browder, The Mothers of Gynecology Statue and Park, 2021, unveiling, https://www.anarchalucybetsey.org/. Educational Use. Accessed February 13, 2023. [This figure appears in color in the online issue]

Juxtaposing the women's pain with their grit and their powerful enduring legacies, Browder adorns each woman with distinctive Afrocentric jewelry—incorporating Ghanaian adinkra symbols and cowry shells. Browder also affords the women dignity and beauty through welded metal in the shape of Black women's hairstyles, including bantu knots, braids, and a small kinky afro. The public monument in Montgomery brings this history (and this article) full circle—powerfully shifting iconographic representation and signifying decades of Black feminist praxis (Figure 3).

## CONCLUSION: LESSONS FOR MEDICAL ANTHROPOLOGY

This article has highlighted how 40 years of Black feminist thought, art, and grassroots activism has changed the pedagogical, cultural, and medical landscapes of the United States. There are implications for the teaching of medical history, medical and health professions training, medical ethics curriculum design, critical health humanities methodologies, reparations debates, and contemporary reproductive justice struggles (especially in the post‐Roe era). While not exhaustive, the efforts highlighted above document an important history of sustained Black feminist praxis, across time and space—with allies and coalitional stakeholders. The article also represents a deliberate scholarly exercise in citing Black women.

Further, the research on which this article is based can be placed in productive conversation with anthropological scholarship on obstetrics, gynecology, and birthing justice. For example, recent important anthropological work has been done on the politics of vesicovaginal fistulas in Niger and Ethiopia (Hannig, 2017; Heller, 2018, on the afterlife of colonial medical violence and women's labor in the Congo (Hunt, 2016), and in ethnographic approaches to intersectional reproductive justice research across various geographic contexts (Bridges, 2011; Davis, 2019; Falu, 2023; Ross & Roberts et al., 2017).

Circling back now to where the article began, I conclude by nodding toward the work of Saidiya Hartman and Deirdre Cooper Owens. Hartman, echoing Nikole Hannah Jones’ *1619 Project*, reminds us: “I too live in the time of slavery, by which I mean I am living in the future created by it” (2007, 133). Cooper Owens reminds us further that “[T]he historical arc of American gynecology resembles other American histories in that it is triumphant. It is a polyphonic narrative that contains the voices of the elite and the downtrodden, and if studied closely, this history evidences how race, class, and gender influenced seemingly value‐neutral fields like medicine” (2017, 3). Through the prism of Black feminism, readers of this *Medical Anthropology Quarterly* special issue can more fully appreciate the transformational truth and reconciliation work done in this area. I have documented Black feminist praxis over the span of four decades now, for Anarcha, Lucy, Betsey, and the unnamed others—and for us all.
